# Sexual function after prostate cancer surgery or radiotherapy in an international registry

**DOI:** 10.1111/bju.70431

**Published:** 2026-08-26

**Authors:** Y. Hess‐Busch, C. Darr, L. Püllen, M. Al‐Nader, M. Wahl, A. V. Bailey, H. Wilhalme, J. Lee, B. Hadaschik, J. Hess, the TrueNTH Global Registry

**Affiliations:** ^1^ Department for Oncology Kliniken Essen‐Mitte Essen Germany; ^2^ Department for Urology University Medicine Essen, University Duisburg‐Essen Essen Germany; ^3^ Department of Urology University Hospital Frankfurt, Goethe University Frankfurt am Main Frankfurt am Main Germany; ^4^ Departments of Urology, OBGYN, & Surgery University of California, Los Angeles (UCLA) Los Angeles CA USA; ^5^ Department of Medicine Statistics Core, David Geffen School of Medicine University of California, Los Angeles (UCLA) Los Angeles CA USA

**Keywords:** prostate cancer, patient‐reported outcomes, sexual function, erectile dysfunction, radical prostatectomy, radiotherapy

## Abstract

**Objectives:**

Our objective was to evaluate patient‐reported sexual function (SF) outcomes and sexual aid use 12 months after curative treatment for prostate cancer (PC).

**Patients & Methods:**

This prospective registry‐based cohort study analysed men with localized or locally advanced PC enrolled in the TrueNTH Global Registry. Patients with baseline and 12‐month patient‐reported outcome measures after radical prostatectomy (RP), external beam radiotherapy (EBRT) ± androgen‐deprivation therapy (ADT), or brachytherapy (BT) were included. SF was assessed using the EPIC‐26 sexual function domain score (SFDS). Baseline and 12‐month EPIC‐26 SFDS were compared by treatment group, with additional analyses among RP patients examining outcomes by intent to nerve spare and sexual aid use. Linear regression, adjusted for age, National Comprehensive Cancer Network risk group, baseline SFDS, and intent to nerve spare was used to identify predictors of 12‐month SFDS among surgery patients.

**Results:**

Overall, 13 413 men were analysed (RP 85.2%, EBRT only 9.1%, EBRT+ADT 4.3%, BT 1.3%). SF declined significantly across all modalities. Median SFDS reduction was greatest after RP (−60.0%) and smallest after BT (−40.2%). At 12 months, poor or absent erectile function was reported by 63.6–91.8% of patients, highest after RP and EBRT+ADT. Sexual aid use increased after treatment but remained low overall. Given the retrospective design and non‐representative sites in an international registry setting, these findings should be interpreted as associational rather than causal because of the potential for unmeasured confounding and cross‐country heterogeneity in treatment selection.

**Conclusion:**

All curative PC treatments were associated with substantial deterioration in SF. These findings support enhanced pre‐treatment counselling and early integration of proactive management and structured sexual rehabilitation into survivorship care.

**Abbreviations**
ADT: androgen deprivation therapy
BT: brachytherapy
EBRT: external beam radiotherapy
ED: erectile dysfunction
EF: erectile function
EPIC: Expanded Prostate Cancer Index Composite
IIEF: International Index of Erectile Function
IQR: interquartile range
MID: minimally important difference
MUSE: medicated urethral system for erection
NCCN: National Comprehensive Cancer Network
ns: nerve‐sparing
PC: prostate cancer
PROM: patient‐reported outcome measure
PSA: prostate specific antigen
RARP: robotic assisted radical prostatectomy
RP: radical prostatectomy
SF: sexual function
SFDS: sexual function domain score
TNGR: True North Global Registry

## Introduction

Prostate cancer (PC) is the second most newly diagnosed malignancy among men worldwide [1] and the second most common cause of cancer‐related deaths in eight of 20 regions worldwide. With more than 375 000 deaths worldwide in 2020, PC is only slightly less significant than lung cancer as a cause of cancer mortality [1]. PC mortality rates have largely declined in most countries [2], probably due to a stage shift related to prostate‐specific antigen testing and improved oncological management [3]. For localized PC, curative treatment options offer excellent long‐term oncological outcomes. These include radical prostatectomy (RP) and radiotherapy (RT), applied as brachytherapy (BT) or external beam radiation therapy (EBRT), with or without androgen‐deprivation therapy (ADT). Large population‐based studies indicate that the 10‐year PC‐specific survival rate for men with favourable localized disease exceeds 98% regardless of treatment modality [4, 5]. The 15‐year PROTEcT trial data showed comparable cancer‐specific survival and overall survival for EBRT with normofractionation and RP, with cancer‐specific death rates of 2.2% (RP) and 2.9% (EBRT), respectively [6].

Although these outcomes are encouraging, they come at a cost: both surgery and R are associated with significant adverse effects, particularly incontinence and erectile dysfunction (ED), which can profoundly affect quality of life [5]. With prolonged recurrence‐free survival after local therapy, attention is increasingly shifting towards individual quality of life and therapy‐associated limitations. In a longitudinal study, ED and satisfaction with sexual life predicted decisional regret in men with local treatment for organ‐confined PC [7]. Furthermore, a relevant number of PC survivors (14%) developed mental health symptoms. Among the most important correlates of poorer mental health were urinary bother, sexual dysfunction, and lower sexual satisfaction [8, 9]. Health‐related quality of life does not seem to differ by intervention [10]. Cross‐country comparisons of functional impairments after definitive treatment of localized PC remain scarce. Given the high prevalence of PC, the potential harms of (over‐)treatment, and quality‐of‐life implications of local therapies, there is an urgent need for real‐world data to inform clinical decision‐making. High‐quality, field‐based evidence is essential to better balance oncologic control with functional outcomes and to guide patients and physicians in choosing the most appropriate management strategy.

The Movember Foundation started the True North Global Registry (TNGR) in 2016 [11]. It is a large initiative aimed at comparing outcomes of localized and locally advanced PC globally to enhance the quality of PC care. The registry's objective is to facilitate international benchmarking and drive quality improvement by collecting standardized clinical and patient‐reported outcome data from participating institutions worldwide.

Studies on erectile function (EF) after PC treatment have predominantly included men with normal or preserved EF at baseline [12, 13], whereas patients with preexisting ED have been less extensively studied. Consequently, evidence regarding functional outcomes among men with ED before PC treatment is limited.

Through further analysis of TNGR data, we aimed to delineate the differences in sexual function (SF) outcomes and what trends were observed in the use of sexual aids across treatment modalities over 12 months in a real‐world setting of patients with PC.

## Methods

TNGR is an international, multicentre, prospective clinical quality registry established to monitor and improve outcomes for men with localized and locally advanced PC. The registry gathered data from more than 200 sites in 16 countries using a standardized study protocol and mandatory data dictionaries. At the time of this study, 92 000 men were enrolled. The registry captures demographic and disease characteristics, treatment information, and follow‐up outcomes.

### Inclusion and Exclusion Criteria

Men were eligible if newly diagnosed with localized or locally advanced PC (any T, any N, M0) and consented to data collection and follow‐up. Exclusion criteria were age <18 years at diagnosis, preexisting ED, acute or chronic conditions significantly limiting study participation, inability to receive study‐relevant information in a language the patient could understand, refusal to participate, or previous definitive PC treatment.

### EPIC‐26

The short version of the Expanded Prostate Cancer Index Composite (EPIC) questionnaire includes 26 questions summarized into five categories (incontinence, irritative/obstructive symptoms, gastrointestinal function, sexuality, and hormonal function), enabling healthcare providers and researchers to evaluate the impact of PC and its treatments on patients' daily lives [14, 15]. Items use 4‐ or 5‐level Likert responses, linearly transformed to a 0–100 scale, with higher scores indicating better outcomes [16]. Minimally important differences have been previously established for each domain, with the minimally important difference for SF specifically being 10–12 points [17]. EPIC‐26 has been validated across the majority of participating countries [18, 19, 20, 21] and enables comparison of outcomes between interventions within TNGR.

### Quality Assurance and Data Governance

The registry ensures data quality through automated validation checks, periodic site audits, and continuous feedback reports. A steering committee comprising clinicians, researchers, and patient representatives oversees ethical data use and strategic direction. All procedures comply with local ethical regulations, and informed consent is obtained from each patient. The study was given ethical approval by Monash University and was conducted in accordance with the local ethical standards and the 2000 revision of the Declaration of Helsinki.

### Statistical Analysis

Baseline patient and clinical characteristics were compared across treatment groups (BT, EBRT + ADT, EBRT only, RP) using chi‐squared tests for categorical variables and Kruskal–Wallis tests for continuous variables. Sexual aid use at baseline and 12 months, and EPIC‐26 sexual function domain scores (SFDS) at the same time intervals were additionally described and compared by treatment group.

Among patients undergoing RP, we further described surgical approach, SFDS, and individual SF question responses at baseline and 12 months, stratified by intent to nerve spare. Responses to individual SF questions at baseline and 12 months were also described by treatment group overall.

To evaluate predictors of 12‐month SF among RP patients, we fit a linear regression model stratified by sexual aid use at baseline and at 12 months. Models were adjusted for age, National Comprehensive Cancer Network (NCCN) risk group, baseline SFDS, and intent to nerve spare. All analyses were performed using SAS version 9.4 (SAS Institute, Cary, NC, USA), with significance set at a two‐sided *P*‐value of <0.05.

## Results

For our analysis, we focused on men diagnosed between 2016 and 2022 with a valid patient‐reported outcome measure (PROM) at baseline and 12 months (but no longer than 15 months) after curative‐intent surgery or RT, resulting in a cohort of 13 413 men (see Fig. 1).

**Fig. 1:**
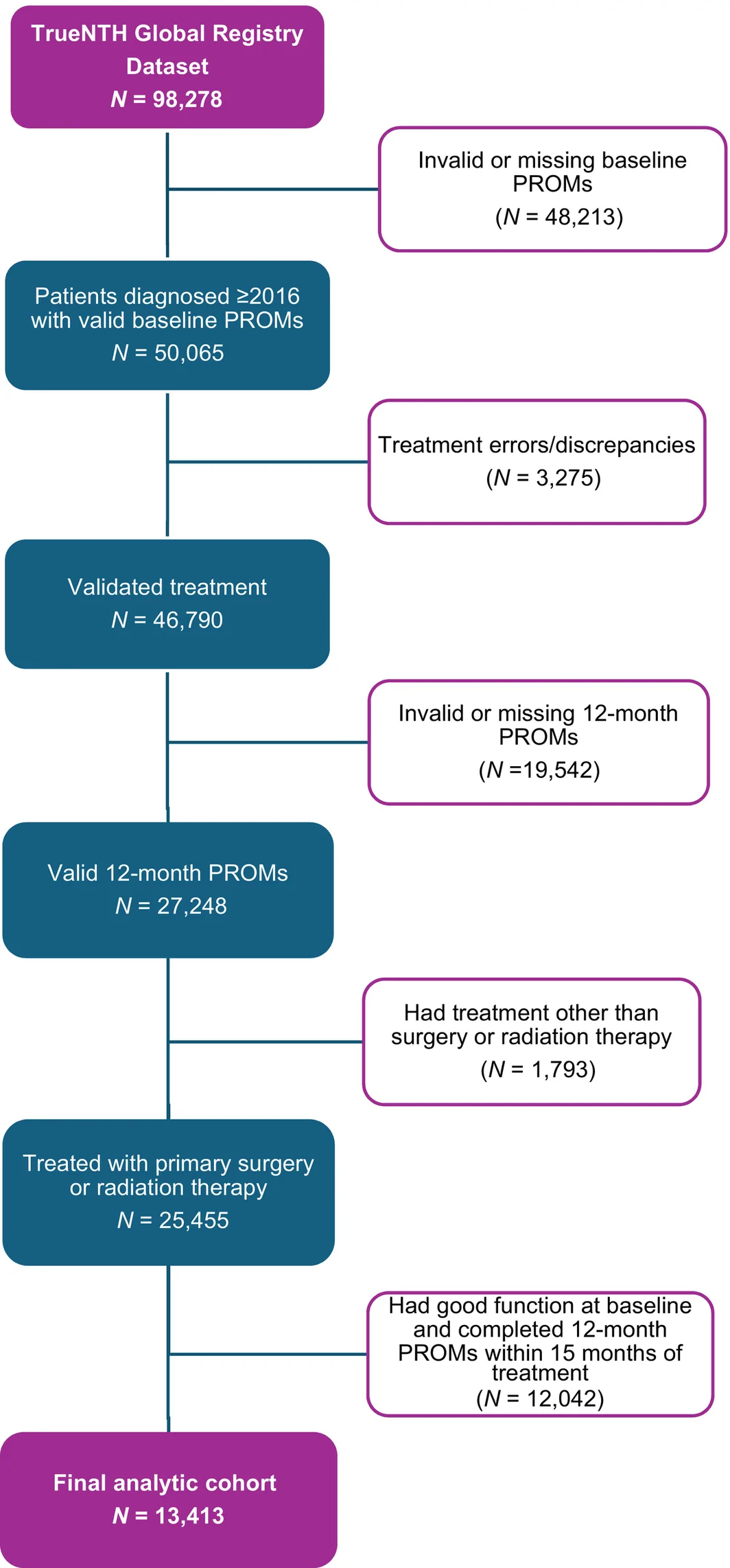
Consort diagram. PROMs, patient‐reported outcome measures.

The RP group was the largest, with 11 429 (85.2%) patients (see Table 1). The EBRT‐only group accounted for 1226 (9.1%), EBRT+ADT for 582 (4.3%), and BT for 176 men (1.3%). The majority of men who had BT had low‐dose rate BT (94.3%). Central Europe included the most patients in all treatment groups (BT 43.8%; EBRT+ADT 26.5%; EBRT only 63.1%; RP 78.8%). To facilitate interpretation in the context of different healthcare settings, participant characteristics stratified by country are provided in Table S1. However, structural characteristics of the participating hospitals were not available.

**Table 1:** Patient characteristics by treatment group.

Characteristic	Treatment				*P*‐value
	BT (*N* = 176)	EBRT + ADT (*N* = 582)	EBRT only (*N* = 1226)	RP (*N* = 11 429)	
Age, years	68 (64–74)	73 (68–77)	74 (68–77)	67 (63–72)	<0.0001*
Country/territory					
Australia/New Zealand	16 (9.1)	52 (8.9)	94 (7.7)	258 (2.3)	<0.0001^†^
Canada	31 (17.6)	197 (33.8)	137 (11.2)	355 (3.1)	
Central Europe	77 (43.8)	154 (26.5)	773 (63.1)	9008 (78.8)	
Hong Kong	0 (0.0)	110 (18.9)	44 (3.6)	155 (1.4)	
Italy	0 (0.0)	9 (1.5)	20 (1.6)	437 (3.8)	
Spain	41 (23.3)	21 (3.6)	121 (9.9)	63 (0.6)	
USA	1 (0.6)	2 (0.3)	0 (0.0)	1047 (9.2)	
United Kingdom	10 (5.7)	37 (6.4)	37 (3.0)	106 (0.9)	
Year of diagnosis					
2016	6 (3.4)	4 (0.7)	14 (1.1)	295 (2.6)	<0.0001^†^
2017	50 (28.4)	87 (14.9)	123 (10.0)	1652 (14.5)	
2018	57 (32.4)	140 (24.1)	280 (22.8)	2413 (21.1)	
2019	38 (21.6)	159 (27.3)	380 (31.0)	3240 (28.3)	
2020	19 (10.8)	130 (22.3)	324 (26.4)	3141 (27.5)	
2021	6 (3.4)	62 (10.7)	105 (8.6)	687 (6.0)	
2022	0 (0.0)	0 (0.0)	0 (0.0)	1 (0.0)	
Comorbidities					
None	77 (48.4)	213 (41.8)	604 (53.7)	6399 (61.9)	<0.0001^†^
1	46 (28.9)	153 (30.0)	254 (22.6)	2688 (26.0)	
≥2	36 (22.6)	144 (28.2)	267 (23.7)	1245 (12.0)	
Missing	17	72	101	1097	
PSA					
0–<4	15 (9.3)	18 (3.3)	75 (6.4)	829 (7.4)	<0.0001^†^
4–<10	129 (79.6)	211 (38.8)	694 (59.7)	6852 (60.8)	
10–<20	17 (10.5)	188 (34.6)	306 (26.3)	2547 (22.6)	
20–<50	1 (0.6)	90 (16.5)	69 (5.9)	840 (7.5)	
≥50	0 (0.0)	37 (6.8)	19 (1.6)	193 (1.7)	
Missing	14	38	63	168	
Clinical T stage					
T0/TX	2 (1.1)	5 (0.9)	6 (0.5)	33 (0.3)	<0.0001^†^
T1	118 (67.0)	215 (37.5)	688 (56.3)	7616 (67.0)	
T2	54 (30.7)	236 (41.1)	438 (35.9)	3239 (28.5)	
T3	2 (1.1)	115 (20.0)	86 (7.0)	467 (4.1)	
T4	0 (0.0)	3 (0.5)	3 (0.2)	12 (0.1)	
Missing	0	8	5	62	
Clinical N stage					
N0/NX	176 (100.0)	552 (96.0)	1190 (97.7)	11 211 (98.9)	<0.0001^†^
N1	0 (0.0)	23 (4.0)	28 (2.3)	127 (1.1)	
Missing	0	7	8	91	
Gleason grade group					
1	94 (53.4)	32 (5.5)	242 (19.8)	2206 (19.5)	<0.0001^†^
2	72 (40.9)	197 (33.9)	531 (43.4)	4223 (37.2)	
3	6 (3.4)	158 (27.2)	263 (21.5)	2380 (21.0)	
4	2 (1.1)	92 (15.8)	97 (7.9)	1606 (14.2)	
5	2 (1.1)	102 (17.6)	91 (7.4)	922 (8.1)	
Missing	0	1	2	92	
NCCN risk group					
Low risk	72 (40.9)	6 (1.0)	146 (11.9)	1428 (12.5)	<0.0001^†^
Favourable intermediate risk	12 (6.8)	12 (2.1)	71 (5.8)	566 (5.0)	
Unfavourable intermediate risk	19 (10.8)	182 (31.3)	417 (34.0)	3347 (29.3)	
High risk	6 (3.4)	271 (46.6)	228 (18.6)	3151 (27.6)	
Very high risk	0 (0.0)	25 (4.3)	25 (2.0)	88 (0.8)	
Regional	0 (0.0)	23 (4.0)	28 (2.3)	127 (1.1)	
Other	67 (38.1)	63 (10.8)	311 (25.4)	2722 (23.8)	

* Kruskal–Wallis *P*‐value.

^†^ Chi‐squared *P*‐value.

Data are presented as median (interquartile range) or *n* (%) unless otherwise indicated.

ADT, androgen‐deprivation therapy; BT, brachytherapy; EBRT, external beam radiotherapy; NCCN, National Comprehensive Cancer Network; PSA, prostate‐specific antigen; RP, radical prostatectomy.

Patients undergoing RP and BT had a lower median age than those receiving EBRT±ADT. A larger percentage of RP patients reported no comorbidities. Most patients had prostate‐specific antigen levels between 4 and 10 ng/mL and organ‐confined disease. Lymph node involvement on conventional imaging was rare in all groups. BT patients mostly had lower‐grade disease and the highest proportion of low‐risk patients according to NCCN risk group, whereas higher‐grade and (very) high‐risk tumours were more common in the EBRT+ADT group.

Baseline SFDS differed significantly (see Table 2). The highest median score was observed in the BT group (44; interquartile range [IQR] 22–58), followed by RP (42; IQR 22–58), EBRT+ADT (31; IQR 17–50), and EBRT only (31; IQR 17–53). Among prostatectomized patients, SF outcomes differed significantly between surgery with intention of nerve sparing (ns) and without (non‐ns) (see Table 3). Patients in the ns group reported a higher SFDS at baseline than those in the non‐ns group (median 47 vs. 35).

**Table 2:** Sexual function characteristics and outcomes by treatment.

Characteristics and outcomes	Treatment				*P*‐value
	BT (*N* = 176)	EBRT and ADT (*N* = 582)	EBRT only (*N* = 1226)	RP (*N* = 11 429)	
Baseline aid use					
No	168 (95.5)	571 (98.1)	1193 (97.3)	11 080 (96.9)	0.2184*
Yes	8 (4.5)	11 (1.9)	33 (2.7)	349 (3.1)	
12‐month aid use					
No	164 (93.2)	576 (99.0)	1186 (96.7)	10 843 (94.9)	<0.0001*
Yes	12 (6.8)	6 (1.0)	40 (3.3)	586 (5.1)	
Baseline sexual function domain score					
Median (IQR)	44 (22–58)	31 (17–50)	31 (17–53)	42 (22–58)	<0.0001^†^
Range	0–88	0–83	0–85	0–92	
12‐month sexual function domain score					
Median (IQR)	26 (17–57)	17 (8–21)	17 (13–35)	17 (5–25)	<0.0001^†^
Range	0–88	0–88	0–100	0–100	

* Chi‐squared *P*‐value.

^†^ Kruskal–Wallis *P*‐value.

Data are presented as *n* (%) unless otherwise indicated.

ADT, androgen‐deprivation therapy; BT, brachytherapy; EBRT, external beam radiotherapy; IQR, interquartile range; RP, radical prostatectomy.

**Table 3:** Baseline and 12‐month sexual function by nerve sparing status.

	Nerve sparing status								
	Missing (*N* = 276)			Non‐nerve sparing (*N* = 4363)			Nerve sparing (*N* = 6790)		
	PROMs time			PROMs time			PROMs time		
	Baseline	12 months	*P*‐value	Baseline	12 months	*P*‐value	Baseline	12 months	*P*‐value
Surgery approach									
Open	52 (20.1)	–	–	1130 (29.5)	–	–	1803 (30.0)	–	–
Laparoscopic	82 (31.7)	–		546 (14.3)	–		486 (8.1)	–	
Robotic	123 (47.5)	–		2130 (55.6)	–		3709 (61.7)	–	
Other	2 (0.8)	–		24 (0.6)	–		14 (0.2)	–	
Missing	17	–		533	–		778	–	
Sexual function domain									
Median (IQR)	45 (22–61)	17 (4–22)	<0.0001^†^	35 (18–53)	13 (4–17)	<0.0001^†^	47 (28–61)	17 (8–32)	<0.0001^†^
Range	0–83	0–79		0–92	0–93		0–92	0–100	
Ability to have an erection									
Very poor to none	61 (22.1)	210 (76.6)	<0.0001*	1386 (31.8)	3790 (88.5)	<0.0001*	1315 (19.4)	4512 (67.1)	<0.0001*
Poor	94 (34.1)	32 (11.7)		1263 (28.9)	332 (7.8)		1731 (25.5)	1250 (18.6)	
Fair	121 (43.8)	27 (9.9)		1714 (39.3)	122 (2.8)		3744 (55.1)	762 (11.3)	
Good	0 (0.0)	4 (1.5)		0 (0.0)	34 (0.8)		0 (0.0)	173 (2.6)	
Very good	0 (0.0)	1 (0.4)		0 (0.0)	5 (0.1)		0 (0.0)	27 (0.4)	
Missing	0	2		0	80		0	66	
Quality of erections									
Very poor to none	76 (28.4)	209 (76.0)	<0.0001*	1427 (33.4)	3499 (82.0)	<0.0001*	1382 (20.7)	4133 (61.7)	<0.0001*
Poor	75 (28.0)	36 (13.1)		1293 (30.3)	558 (13.1)		1840 (27.6)	1514 (22.6)	
Fair	92 (34.3)	26 (9.5)		1362 (31.9)	163 (3.8)		2921 (43.8)	823 (12.3)	
Good	22 (8.2)	4 (1.5)		179 (4.2)	39 (0.9)		509 (7.6)	208 (3.1)	
Very good	3 (1.1)	0 (0.0)		11 (0.3)	6 (0.1)		22 (0.3)	24 (0.4)	
Missing	8	1		91	98		116	88	
Sexual function bother									
No problem	69 (25.4)	101 (36.7)	<0.0001*	1291 (30.1)	1223 (28.8)	<0.0001*	1485 (22.2)	1194 (17.8)	<0.0001*
Very small problem	41 (15.1)	32 (11.6)		746 (17.4)	574 (13.5)		1252 (18.7)	893 (13.3)	
Small problem	73 (26.8)	35 (12.7)		954 (22.3)	567 (13.3)		1848 (27.6)	1223 (18.2)	
Moderate problem	49 (18.0)	36 (13.1)		796 (18.6)	774 (18.2)		1499 (22.4)	1630 (24.3)	
Big problem	40 (14.7)	71 (25.8)		496 (11.6)	1114 (26.2)		604 (9.0)	1762 (26.3)	
Missing	4	1		80	111		102	88	

* Chi‐squared *P*‐value.

^†^ Wilcoxon rank sum *P*‐value.

Data are presented as *n* (%) unless otherwise indicated.

IQR, interquartile range; PROMs, patient‐reported outcomes measures.

At 12 months, EPIC‐26 SFDS declined across all treatment groups. The greatest preservation of SF was seen in the BT group (median 26, Δ −40.2%). In all other groups, the score fell to a median of 17 (EBRT+ADT: Δ −46.7%; EBRT only: Δ −45.3% and RP: Δ −60.0%). Median SFDS decreased within 1 year from 35 to 13 in the non‐ns group (Δ −64.0%; *P* < 0.0001) and from 47 to 17 in the ns group (Δ −64.6%; *P* < 0.0001).

None of the patients initially reported a *‘*good’ or ‘very good’ ability to have an erection (see Table 4). At baseline, 50.5% (BT), 66.0% (EBRT+ADT), 63.5% (EBRT only) and 51.2% (RP: 60.7% non‐ns and 44.9% ns) rated their ability to have an erection as ‘poor’ or *‘*very poor to none’. After 12 months, these rates increased notably to 63.6% (BT), 91.8% (EBRT+ADT), 81.8% (EBRT only), and 89.8% (RP), respectively. Among surgical patients, 96.3% (non‐ns) and 85.7% (ns) reported EF in the ‘poor’ to ‘very poor to none’ range after 1 year (Δ + 58.7% vs. Δ +91.9%). At that time, ‘good’ or ‘very good’ EF remained low across all groups: 6.4% (BT), 1.4% (EBRT+ADT), 4.0% (EBRT only), and 2.2% (RP).

**Table 4:** Baseline and 12‐month sexual function by treatment.

	Treatment											
	BT (*N* = 176)			EBRT and ADT (*N* = 582)			EBRT only (*N* = 1226)			RP (*N* = 11 429)		
	PROMs time			PROMs time			PROMs time			PROMs time		
	Baseline	12 months	*P*‐value	Baseline	12 months	*P*‐value	Baseline	12 months	*P*‐value	Baseline	12 months	*P*‐value
Ability to have an erection												
Very poor to none	43 (24.4)	64 (37.0)	<0.0001*	220 (37.8)	428 (75.1)	<0.0001*	488 (39.8)	719 (60.3)	<0.0001*	2762 (24.2)	8512 (75.5)	<0.0001*
Poor	46 (26.1)	46 (26.6)		164 (28.2)	95 (16.7)		291 (23.7)	256 (21.5)		3088 (27.0)	1614 (14.3)	
Fair	87 (49.4)	52 (30.1)		198 (34.0)	39 (6.8)		447 (36.5)	170 (14.3)		5579 (48.8)	911 (8.1)	
Good	0 (0.0)	11 (6.4)		0 (0.0)	7 (1.2)		0 (0.0)	44 (3.7)		0 (0.0)	211 (1.9)	
Very good	0 (0.0)	0 (0.0)		0 (0.0)	1 (0.2)		0 (0.0)	3 (0.3)		0 (0.0)	33 (0.3)	
Missing	0	3		0	12		0	34		0	148	
Quality of erections												
Very poor to none	51 (29.3)	79 (45.9)	0.0062*	206 (37.1)	432 (76.9)	<0.0001*	486 (40.5)	711 (59.9)	<0.0001*	2885 (25.7)	7841 (69.7)	<0.0001*
Poor	39 (22.4)	31 (18.0)		177 (31.8)	92 (16.4)		320 (26.7)	264 (22.2)		3208 (28.6)	2108 (18.8)	
Fair	73 (42.0)	49 (28.5)		150 (27.0)	30 (5.3)		349 (29.1)	174 (14.7)		4375 (39.0)	1012 (9.0)	
Good	9 (5.2)	13 (7.6)		23 (4.1)	7 (1.2)		42 (3.5)	36 (3.0)		710 (6.3)	251 (2.2)	
Very good	2 (1.1)	0 (0.0)		0 (0.0)	1 (0.2)		2 (0.2)	2 (0.2)		36 (0.3)	30 (0.3)	
Missing	2	4		26	20		27	39		215	187	
Sexual function bother												
No problem	64 (36.4)	46 (26.6)	0.0171*	220 (38.7)	230 (41.0)	<0.0001*	476 (39.6)	434 (36.5)	<0.0001*	2845 (25.3)	2518 (22.4)	<0.0001*
Very small problem	27 (15.3)	24 (13.9)		86 (15.1)	77 (13.7)		180 (15.0)	159 (13.4)		2039 (18.1)	1499 (13.3)	
Small problem	31 (17.6)	34 (19.7)		94 (16.5)	53 (9.4)		231 (19.2)	183 (15.4)		2875 (25.6)	1825 (16.3)	
Moderate problem	46 (26.1)	44 (25.4)		98 (17.2)	78 (13.9)		181 (15.1)	215 (18.1)		2344 (20.8)	2440 (21.7)	
Big problem	8 (4.5)	25 (14.5)		71 (12.5)	123 (21.9)		134 (11.1)	199 (16.7)		1140 (10.1)	2947 (26.2)	
Missing	0	3		13	21		24	36		186	200	

* Chi‐square *P*‐value.

Data are presented as *n* (%) unless otherwise indicated.

ADT, androgen‐deprivation therapy; BT, brachytherapy; EBRT, external beam radiotherapy; PROMs, patient‐reported outcomes measures; RP, radical prostatectomy.

Similarly, 51.7% (BT), 68.9% (EBRT+ADT), 67.2% (EBRT only), and 54.3% (RP) rated erection quality as ‘poor’ or ‘very poor to none’ at baseline. At 12 months, these figures remained unfavourable, at 63.9% (BT), 93.3% (EBRT+ADT), 82.1% (EBRT only), and 88.5% (RP).

Patient‐reported bother related to SF varied significantly across treatment groups and changed notably from baseline to 12 months (see Table 4). RP showed the greatest increase in severe sexual bother, and BT showed the smallest shift. In the RP group, patients reporting a ‘big problem’ increased from 10.1% at baseline to 26.2% at 12 months (*P* < 0.0001). Significant increases were also observed in the BT (4.5–14.5%) and EBRT+ADT (12.5–21.9%) groups and to a slightly lesser extent in the EBRT‐only group (11.1–16.7%). The proportion reporting ‘no problems’ decreased in all groups (BT: 36.4–26.6%; EBRT only: 39.6–36.5%, RP: 25.3–22.4%), except EBRT+ADT, where it increased slightly from 38.7–41.0%.

The use of sexual aids represents a clinically relevant component of conservative management of ED. Sexual aid use was defined as the utilization of any form of sexual aid (i.e., oral medication, medicated urethral system for erection, penile injection, vacuum erection devices, or other aid, device, or medication). At baseline, use was generally low, ranging from 1.9% in the EBRT+ADT group to 4.5% in the BT group, without statistically significant differences between groups (*P* = 0.2184) (see Table 2). After 12 months, aid use increased in all groups except the EBRT+ADT group (range 1.0–6.8%). RP showed the greatest increase (64.5%) within 1 year, although overall use remained low. Increases of 51.1% and 22.2% were observed in BT and EBRT only, respectively, whereas use decreased by 47.4% in the EBRT+ADT group. Differences between groups were statistically significant (*P* < 0.0001).

Multivariate regression was not possible in RT groups when aid use was considered due to limited subgroup numbers. In RP patients, several factors were significantly associated with 12‐month EPIC‐26 SFDS, with patterns differing by aid use (see Table 5). In this analysis, No‐No was defined as patients who neither used sexual aids at baseline nor after 12 months and Yes‐No represented men who stopped using aids after baseline. Higher baseline SFDS was consistently associated with better 12‐month outcomes. Patients already using aids at baseline had poorer function at 12 months than non‐users. Increasing age was significantly associated with lower SF in among those not using aids 1 year after surgery but not in other groups. Higher NCCN risk groups tended to predict worse outcomes, particularly in the No‐No group, where unfavourable intermediate‐risk, high‐risk and very high‐risk were independently associated with lower 12‐month SF. Intention to perform ns was a strong positive predictor in the No‐No (β = 5.25; *P* < 0.01) and Yes‐No (β = 6.63; *P* < 0.01) groups, with the largest, though non‐significant, effect in the Yes‐Yes group (β = 14.86). Overall, baseline SFDS, age, cancer risk category and intention to ns emerged as key determinants of post‐prostatectomy SF, with their influence varying by sexual aid use.

**Table 5:** Multivariate linear regression predicting 12‐month EPIC‐26 sexual function stratified on aid use at baseline and 12 months in radical prostatectomy patients.

	Aid use at baseline and 12 months			
	No‐no (*N* = 9911)	No‐yes (*N* = 480)	Yes‐no (*N* = 254)	Yes‐yes (*N* = 80)
	β (SE)	β (SE)	β (SE)	β (SE)
Constant	13.94 (1.95)**	23.56 (12.17)	30.15 (10.40)**	11.51 (23.11)
Age, per every 10 years	−0.88 (0.27)**	1.25 (0.59)	−4.92 (1.49)**	1.40 (3.27)
NCCN risk group				
Low risk	Ref.	Ref.	Ref.	Ref.
Favourable intermediate risk	−1.57 (0.87)	5.68 (5.67)	5.89 (4.32)	−4.68 (12.43)
Unfavourable intermediate risk	−1.40 (0.56)*	−5.49 (3.34)	6.14 (2.75)*	−14.36 (7.50)
High risk	−3.08 (0.57)**	−4.75 (3.79)	3.29 (3.08)	−4.28 (8.02)
Very high risk	−5.30 (1.92)**	14.72 (17.73)	1.48 (8.68)	–
Regional	−4.01 (1.62)*	9.01 (9.03)	6.26 (10.69)	–
Other	−0.11 (0.57)**	−8.73 (3.30)**	2.20 (2.90)	−1.19 (7.01)
Baseline sexual function EPIC‐26 domain score, per every 10 points	2.03 (0.08)**	2.32 (0.59)**	1.86 (0.48)**	1.30 (1.43)
Intent to nerve spare				
No	Ref.	Ref.	Ref.	Ref.
Yes	5.25 (0.35)**	3.79 (2.98)	6.66 (2.10)**	14.86 (8.77)

* *P* < 0.05.

** *P* < 0.01.

EPIC‐26, Expanded Prostate Cancer Index Composite; NCCN, National Comprehensive Cancer Network; Ref, reference; SE, standard error.

## Discussion

This study presents a large, multi‐national cohort of men with PC treated with curative intent, offering important insights into real‐world SF outcomes after radical RP or RT‐based treatments. Our findings confirm and expand upon existing literature by quantifying the magnitude of SF decline over the first postoperative year across different treatment modalities and exploring the modifying role of sexual aid use.

In our study, the EPIC‐26 SFDS with values between 31 and 58 at baseline indicated poor SF in all subgroups. Comparable baseline values have been reported in men undergoing RP or RT for localized PC [9, 22]. The CEASAR‐Study further contextualized these scores, demonstrating that an SFDS <80 already indicates impaired SF. Only 2% of men with values between 41 and 60 reported very good SF [16]. Psychological distress related to PC diagnosis and workup may transiently impair EF, potentially leading to an underestimation of baseline SF [23, 24].

BT was associated with the most favourable sexual outcomes, with patients retaining the highest median SFDS at 12 months. However, these patients were younger, had lower‐risk disease, and highest baseline SFDS. These findings align with CaPSURE data showing superior erectile outcomes after BT compared with EBRT and RP [25, 26, 27]. Although this may reflect the more localized nature of BT and its reduced collateral damage to neurovascular structures, it is also likely influenced by favourable baseline characteristics and patient selection in this cohort.

Other RT modalities showed lower baseline SFDS but similar relative declines over time. Additional ADT use is likely to worsen SF because of the systemic androgen deprivation [28, 29]. However, in our cohort, the difference between the EBRT‐only and EBRT+ADT groups was small. This may reflect differences in tumour characteristics, patient selection, and variability in ADT timing and duration, which could not be assessed in our study.

With regard to EF, no patient reported a ‘good’ or ‘very good’ ability to have an erection at baseline. The additional use of ADT led to a greater increase than EBRT only or BT but was still significantly lower than in patients who had undergone RP. Patients in the ProtecT‐study who received EBRT+ADT reported an increase in ED 1 year after radiation [30].

The small difference between the EBRT‐only and EBRT+ADT groups could possibly be explained by the timing of ADT administration and the composition of the cohort. In patients with intermediate‐risk PC, the RTOG 9910 trial confirmed no additive benefit of ADT beyond 4 months [31]. In patients with intermediate‐risk PC suitable for EBRT+ADT, EBRT in combination with short‐term ADT (4–6 months) is recommended [32]. Despite clear recommendations for ADT use in EBRT, the median duration of ADT use ranged from 9 to 32 months in high‐risk PC [32]. In the ProtecT‐study, men with localized PC and EBRT received neoadjuvant ADT 3–6 months before and during irradiation [6]. Unfortunately, in our study, we were unable to check the setting of ADT use (neoadjuvant, concurrent, or adjuvant) and the timing of the PROMs in relation to ADT use, so this could have some impact.

RP patients rated their overall SF as comparable to that of the BT cohort at baseline and exhibited the greatest median decline in the EPIC‐26 SFDS at 12 months. Population‐based analyses have shown that RP is consistently associated with larger early declines in SF than EBRT, despite some late convergence of domain scores [33]. A rapid initial deterioration was also reported in the CEASAR study within the first 6–12 months, although not to the same extent [16]. In the MUSIC statewide collaborative EPIC‐26, SFDS dropped from 71 at baseline to 22 1 year after RP [34]. In another study, the EPIC‐26 SFDS 12 months after surgery ranged between 21 (non‐ns) and 35 (bilateral ns), with a further minimal increase after 24 months reaching values between 23 (non‐ns) and 39 (bilateral ns) [22]. Similarly, SFDS 1 year after RP was 23 (open RP) and 30 (robotic‐assisted RP, RARP), respectively [35]. Patients with non‐metastatic PC who had completed their primary treatment at least 3–6 months before assessment and had at least one additional risk factor for ED had a median SFDS of 21 [36]. Despite the high proportion of ns surgeries, nearly 90% of men in our study reported poor or no EF at 12 months. Although older studies reported a much clearer difference between ns‐ and non‐ns‐surgical techniques in terms of preserving EF [37], more recent studies using PROMs consistently show a high incidence of ED after RP, even with nerve preservation [35, 38]. At 12 months, mean International Index of Erectile Function (IIEF)‐5 scores were significantly higher in the NeuroSAFE group than in the standard RARP group (12.7 vs. 9.7; *P* < 0.0001) [39]. Still, only 20% of patients in the NeuroSAFE group, compared with 14% of patients in the standard group, had an IIEF‐5 score > 21 at 1 year after surgery. Since our data were collected in over 200 centres with a varying quality of nerve preservation, surgical factors and the relationship between case load and outcome may explain the only slight difference in outcomes between ns‐ and non‐ns‐RP.

SF may continue to evolve beyond 12 months after PC treatment. Long‐term data from the ProtecT trial demonstrated treatment‐specific trajectories of SF over 6 years, with some recovery following the initial deterioration after RP and RT [40]. Longitudinal EPIC‐26 data demonstrated changes in SF extending well beyond 12 months after prostatectomy [41]. Longer follow‐up may capture not only continued physiological recovery from treatment‐related injury but also the time required for patients to recognize persistent ED, seek professional support, and initiate ED treatment. Consequently, our 12‐month assessment should be interpreted as an intermediate functional outcome rather than a measure of definitive sexual recovery.

Patients with good potency at baseline who underwent RARP consistently showed improved potency after surgery [42]. O'Neil et al. found a strong relationship between baseline function and function at later timepoints, indicating that RARP can preserve SF in patients with higher preoperative function [43]. The higher baseline score in the ns group reflects a possible selection bias because, in routine clinical practice, the decision to perform ns surgery often depends on tumour characteristics and pre‐existing EF. Although the baseline values of the EPIC‐26 SFDS in our study were higher in the RARP subgroup than in the open RP group, the values were still generally low. More precise dissection techniques of the neurovascular bundle could improve the results of EF. A systematic review and meta‐analysis including seven nonrandomized studies reporting on EF all showed statistically significantly higher EF rates in the NeuroSAFE group [44]. Very recently, Dinneen et al. confirmed that robotic‐assisted precise dissection of the neurovascular bundle resulted in an adjusted mean difference of 3.18 points of the IIEF‐5 score [39]. Whether these differences make a clinical difference for the individual man with treatment‐associated ED is questionable at this score level [16].

In 2015, the International Consultation for Sexual Medicine recommendations stated that there are conflicting data as to whether penile rehabilitation with phosphodiesterase‐5 inhibitors improve EF after RP [45]. However, the absence of postoperative erections in the early period is associated with unsatisfactory cavernous oxygenation. This can cause fibrosis of the corpora cavernosa and lead to veno‐occlusive dysfunction, favouring an early intervention [46]. Despite the high prevalence of post‐treatment ED in all groups, the use of sexual aids remained low.

In our study, RP was associated with the highest increase in sexual bother over time. The ceiling/floor effect provides a good explanation: those who start off higher have more to lose. RP patients were among those with the highest baseline SFDS scores and the highest percentage decline.

In addition, men who received EBRT ± ADT were significantly older at baseline, and sexual activity may have been less important because of their age. Sprangers et al. explained similar findings by the possibility that many survivors undergo a ‘response shift’, in which their perception of quality of life and treatment‐related distress changes as they prioritize survival over symptoms or physical limitations experienced [47]. The willingness to prepare patients for possible non‐oncological side effects of the therapy (such as ED) may be less evident in a surgical speciality. Derogar et al. found that preparedness for urinary problems or sexual side effects before surgery decreased bother from urinary incontinence and ED 1 year after surgery [48]. It is also possible that the results represent patients' wishes for social desirability rather than the real situation. However, this cannot be verified retrospectively.

The primary purpose of the stratified sexual‐aid analysis was to characterize the use of sexual aids across PC treatment modalities and not to establish their efficacy. At baseline, aid use was low in all treatment groups. Although overall aid use remained low, its increase was most pronounced in the RP and BT groups and decreased in the EBRT‐only group. Men in the RP and BT group had higher SFDS, lower proportion of ‘poor’ and ‘very poor’ erection and lower age at baseline, and men with RP had the highest percentage loss of EF, which may account for a greater demand to restore SF. Preoperative preparedness for sexual side effects may also motivate men to actively seek treatment in the case of postoperative ED [48, 49]. Interestingly, patients who stopped using aids after baseline (Yes‐No group) had poorer outcomes than those who had never used them, suggesting that discontinuation may reflect unmet expectations or lack of efficacy. In the Yes‐No group, unfavourable intermediate risk was associated with improved outcomes, though this counterintuitive finding may reflect unmeasured confounding or small subgroup size. The use of sexual aids may be associated with subsequent SF; however, interpretation of such an association is complicated by confounding by indication, as patients with greater ED may be more likely to use sexual aids.

In multivariate analyses limited to RP patients, baseline SF, age, NCCN risk group, and ns intent were all independently associated with 12‐month SF. These factors varied in their predictive strength depending on patterns of aid use, highlighting the complexity of post‐treatment sexual health and the need for personalized rehabilitation strategies. The inability to conduct multivariate analyses in radiation‐treated patients because of the limited number of aid users suggests a need for broader implementation of PROMs to capture and support strategies in these populations.

From a clinical perspective, these findings highlight the considerable negative impact of all treatment modalities on SF and EF over time. This study reinforces the importance of pre‐treatment counselling tailored to the chosen modality and individual patient characteristics. Patients should be informed about the potential for ED, the expected course of recovery, the role of intention to ns, and the availability and limitations of sexual aids and rehabilitation. Moreover, the variability in outcomes among aid users highlights the need for proactive management and structured support programs to optimize long‐term sexual health.

### Strengths and Limitations

Although our study was conducted retrospectively, it reflects real‐world data of a substantial number of men with PC. However geographic and comorbidity patterns emerged: Central European centres contributed the majority of patients and may represent high‐volume centres with established multidisciplinary care pathways. Further, despite representation from a large international cohort, included sites for some countries were highly selected, which introduces additional potential for measurement heterogeneity and residual confounding that adjustment cannot fully address. Second, men in the RP and BT groups were younger and healthier at baseline, reflecting selection biases that must be accounted for in comparative‐effectiveness studies. Our study was designed as a retrospective cross‐sectional study and thus only allows a snapshot of SF at fixed time points. Additionally, our follow‐up period of 12 months limits our ability to fully characterize the long‐term implications of treatment on SF and recovery. These findings should be interpreted as associational rather than causal and as hypothesis generation for future prospective – ideally randomized – comparative studies.

## Conclusion

In this large, international cohort of men treated with curative intent for PC, significant declines in SF and increases in patient‐reported bother were observed across all treatment modalities, especially after RP. Although sexual aid use increased after treatment, it remained low overall and varied in effectiveness depending on patient profile. These findings highlight the importance of early, individualized counselling and the integration of structured sexual rehabilitation into survivorship care to support quality of life following PC treatment.

## Funding

This work was supported by Movember. The analysis and interpretation in the manuscript are those of the authors and not of the TrueNTH Global Registry or Movember.

## Disclosure of Interests

The authors declare no conflict of interest.

## Supporting information


**Table S1.** Patient characteristics by country/territory.

## Data Availability

The data that support the findings of this study are available from the corresponding author upon reasonable request.
