# Supplementary material for: Sexual function after prostate cancer surgery or radiotherapy in an international registry
Source: BJU Int. 2026 Aug 26;138(6):1092–103. doi: 10.1111/bju.70431 (PMC13741107; doi:10.1111/bju.70431)
Supplement: Supplementary file 1 [file BJU-138-1092-s001.pdf]

Supplementary Table 1: Patient characteristics by country/territory

	Country/Territory								
	Australia/New Zealand (N=420)	Canada (N=720)	Central Europe (N=10012)	Hong Kong (N=309)	Italy (N=466)	Spain (N=246)	USA (N=1050)	United Kingdom (N=190)	P-value
**Age, Median (IQR)**	68.0 (64.0, 71.0)	67.0 (63.0, 72.0)	68.0 (64.0, 73.0)	70.0 (66.0, 74.0)	67.0 (63.0, 71.0)	71.0 (65.0, 75.0)	66.0 (61.0, 70.0)	68.0 (64.0, 73.0)	<.0001[1]
**Year of Diagnosis**, n (%)									<.0001[2]
2016	0 (0.0%)	0 (0.0%)	277 (2.8%)	0 (0.0%)	23 (4.9%)	7 (2.8%)	12 (1.1%)	0 (0.0%)	
2017	95 (22.6%)	179 (24.9%)	1227 (12.3%)	4 (1.3%)	61 (13.1%)	54 (22.0%)	291 (27.7%)	1 (0.5%)	
2018	104 (24.8%)	201 (27.9%)	1857 (18.5%)	81 (26.2%)	145 (31.1%)	86 (35.0%)	284 (27.0%)	132 (69.5%)	
2019	110 (26.2%)	146 (20.3%)	3038 (30.3%)	94 (30.4%)	134 (28.8%)	64 (26.0%)	209 (19.9%)	22 (11.6%)	
2020	74 (17.6%)	90 (12.5%)	3102 (31.0%)	94 (30.4%)	57 (12.2%)	34 (13.8%)	156 (14.9%)	7 (3.7%)	
2021	37 (8.8%)	104 (14.4%)	511 (5.1%)	36 (11.7%)	46 (9.9%)	1 (0.4%)	97 (9.2%)	28 (14.7%)	
2022	0 (0.0%)	0 (0.0%)	0 (0.0%)	0 (0.0%)	0 (0.0%)	0 (0.0%)	1 (0.1%)	0 (0.0%)	
**Number of Comorbidities**, n (%)									<.0001[2]
None	103 (27.3%)	353 (64.4%)	5807 (64.6%)	61 (19.7%)	174 (38.2%)	1 (0.5%)	741 (71.2%)	53 (27.9%)	
1	132 (35.0%)	150 (27.4%)	2287 (25.4%)	98 (31.7%)	214 (47.0%)	60 (28.0%)	128 (12.3%)	72 (37.9%)	
2+	142 (37.7%)	45 (8.2%)	898 (10.0%)	150 (48.5%)	67 (14.7%)	153 (71.5%)	172 (16.5%)	65 (34.2%)	
Missing	43	172	1020	0	11	32	9	0	
**PSA**, n (%)									<.0001[2]
0-<4	34 (8.4%)	24 (3.6%)	739 (7.4%)	8 (2.8%)	32 (6.9%)	7 (3.6%)	83 (8.6%)	10 (6.3%)	
4-<10	256 (63.4%)	384 (57.8%)	5937 (59.4%)	104 (36.6%)	302 (64.9%)	127 (65.5%)	678 (70.5%)	98 (62.0%)	
10-<20	88 (21.8%)	196 (29.5%)	2339 (23.4%)	96 (33.8%)	95 (20.4%)	46 (23.7%)	161 (16.7%)	37 (23.4%)	
20-<50	25 (6.2%)	53 (8.0%)	782 (7.8%)	52 (18.3%)	31 (6.7%)	9 (4.6%)	38 (4.0%)	10 (6.3%)	
50+	1 (0.2%)	7 (1.1%)	202 (2.0%)	24 (8.5%)	5 (1.1%)	5 (2.6%)	2 (0.2%)	3 (1.9%)	
Missing	16	56	13	25	1	52	88	32	
**Clinical T Stage**, n (%)									<.0001[2]
T0/TX	4 (1.0%)	12 (1.7%)	4 (0.0%)	0 (0.0%)	0 (0.0%)	4 (1.6%)	21 (2.0%)	1 (0.6%)	
T1	253 (63.7%)	265 (37.6%)	6766 (67.6%)	225 (72.8%)	163 (35.2%)	102 (41.5%)	839 (81.7%)	24 (13.3%)	
T2	123 (31.0%)	323 (45.9%)	2830 (28.3%)	67 (21.7%)	285 (61.6%)	99 (40.2%)	152 (14.8%)	88 (48.9%)	
T3	17 (4.3%)	99 (14.1%)	401 (4.0%)	16 (5.2%)	15 (3.2%)	41 (16.7%)	15 (1.5%)	66 (36.7%)	
T4	0 (0.0%)	5 (0.7%)	11 (0.1%)	1 (0.3%)	0 (0.0%)	0 (0.0%)	0 (0.0%)	1 (0.6%)	
Missing	23	16	0	0	3	0	23	10	
**Clinical N Stage**, n (%)									<.0001[2]
N0/NX	408 (97.4%)	697 (98.9%)	9904 (98.9%)	299 (96.8%)	390 (93.3%)	243 (98.8%)	1014 (99.6%)	174 (96.7%)	
N1	11 (2.6%)	8 (1.1%)	108 (1.1%)	10 (3.2%)	28 (6.7%)	3 (1.2%)	4 (0.4%)	6 (3.3%)	
Missing	1	15	0	0	48	0	32	10	
**Gleason Grade Group**, n (%)									<.0001[2]
GG 1	54 (12.9%)	40 (5.6%)	1998 (20.0%)	86 (27.8%)	158 (34.1%)	81 (32.9%)	146 (15.1%)	11 (5.8%)	
GG 2	203 (48.3%)	386 (53.8%)	3671 (36.7%)	91 (29.4%)	107 (23.1%)	70 (28.5%)	402 (41.6%)	93 (49.2%)	
GG 3	84 (20.0%)	174 (24.3%)	2115 (21.1%)	33 (10.7%)	83 (17.9%)	45 (18.3%)	217 (22.5%)	56 (29.6%)	
GG 4	41 (9.8%)	46 (6.4%)	1408 (14.1%)	52 (16.8%)	91 (19.6%)	29 (11.8%)	115 (11.9%)	15 (7.9%)	
GG 5	38 (9.0%)	71 (9.9%)	815 (8.1%)	47 (15.2%)	25 (5.4%)	21 (8.5%)	86 (8.9%)	14 (7.4%)	
Missing	0	3	5	0	2	0	84	1	
**NCCN Risk Group**, n (%)									<.0001[2]
Low Risk	40 (9.5%)	18 (2.5%)	1299 (13.0%)	39 (12.6%)	93 (20.0%)	45 (18.3%)	116 (11.0%)	2 (1.1%)	
Favorable Intermediate Risk	11 (2.6%)	12 (1.7%)	530 (5.3%)	31 (10.0%)	38 (8.2%)	17 (6.9%)	20 (1.9%)	2 (1.1%)	
Unfavorable Intermediate Risk	115 (27.4%)	266 (36.9%)	3025 (30.2%)	48 (15.5%)	124 (26.6%)	52 (21.1%)	280 (26.7%)	55 (28.9%)	
High Risk	94 (22.4%)	171 (23.8%)	2784 (27.8%)	126 (40.8%)	126 (27.0%)	59 (24.0%)	229 (21.8%)	67 (35.3%)	
Very High Risk	1 (0.2%)	26 (3.6%)	85 (0.8%)	4 (1.3%)	0 (0.0%)	12 (4.9%)	0 (0.0%)	10 (5.3%)	
Regional	11 (2.6%)	8 (1.1%)	108 (1.1%)	10 (3.2%)	28 (6.0%)	3 (1.2%)	4 (0.4%)	6 (3.2%)	

Other	148 (35.2%)	219 (30.4%)	2181 (21.8%)	51 (16.5%)	57 (12.2%)	58 (23.6%)	401 (38.2%)	48 (25.3%)	

[1]Kruskal-Wallis p-value; [2]Chi-Square p-value;
